# Revisiting Metal Toxicity in Neurodegenerative Diseases and Stroke: Therapeutic Potential

**DOI:** 10.14437/NRTOA-1-107

**Published:** 2014-12-31

**Authors:** Joy Mitra, Velmarini Vasquez, Pavana M Hegde, Istvan Boldogh, Sankar Mitra, Thomas A Kent, Kosagi S Rao, Muralidhar L Hegde

**Affiliations:** 1 Department of Radiation Oncology, Houston Methodist Research Institute; 2 Houston Methodist Neurological Institute; 3 Institute of Academic Medicine, Houston Methodist Hospital, Houston Texas 77030, USA; 4 Weill Cornell Medical College of Cornell University, New York, NY 10065, USA; 5 Centre for Neuroscience, Institute for Scientific Research and Technology Services (INDICASAT-AIP), City of Knowledge, Republic of Panama; 6 Department of Biotechnology, Acharya Nagarjuna University, Guntur, India; 7 Department of Microbiology and Immunology, University of Texas Medical Branch, Galveston, Texas 77555, USA; 8 Department of Neurology, Baylor College of Medicine and Center for Translational Research on Inflammatory Diseases Michael E. DeBakey Veterans Affairs Medical Center, Houston, Texas 77030, USA

## Abstract

Excessive accumulation of pro-oxidant metals, observed in affected brain regions, has consistently been implicated as a contributor to the brain pathology including neurodegenerative diseases and acute injuries such as stroke. Furthermore, the potential interactions between metal toxicity and other commonly associated etiological factors, such as misfolding/aggregation of amyloidogenic proteins or genomic damage, are poorly understood. Decades of research provide compelling evidence implicating metal overload in neurological diseases and stroke. However, the utility of metal toxicity as a therapeutic target is controversial, possibly due to a lack of comprehensive understanding of metal dyshomeostasis-mediated neuronal pathology. In this article, we discuss the current understanding of metal toxicity and the challenges associated with metal-targeted therapies.

## Metal accumulation in the brain, a common phenomenon in the pathophysiology of neurodegenerative diseases and stroke

The optimum levels of trace elements and their homeostasis in individual organs are essential for maintaining vital functions. Nutritional deficiencies and metabolic disorders exhibit potential cause-and-effect relationship in many pathological conditions. Although many metal ions, some at trace levels, are essential for life, excess accumulation can be highly toxic and possibly fatal. Neurodegeneration is believed to be the most common manifestation of metal toxicity. For example, etiological link between abnormal metal accumulation in the brain and aging or various neurological disorders, including Alzheimer’s Disease (AD), Parkinson’s Disease (PD), Amyotrophic Lateral Sclerosis (ALS), Wilson’s Disease (WD), and stroke was observed [1–7]. Acute metal toxicity is implicated in stroke and certain conditions that result from hereditary defects in the regulation of metal homeostasis. For example, the dysregulation of metal ions due to the acute release of free iron (Fe) following hemorrhagic stroke causes massive neuronal injury [3,8]. Furthermore, neurotoxicity from acute increase in the level of zinc (Zn) and other transition metals may play a critical role after ischemic focal brain injury [9].

### Environmental/dietary sources versus genetic factors

Fe accumulation in the brain during hemorrhagic stroke is thought to be due to the breakdown of hemoglobin. However, the source and the mechanisms of accumulation of other metals in the brain are unclear, because only in a few cases, this could be linked to dietary or occupational exposures [10]. It is generally believed that both environmental and genetic factors are responsible for abnormal metal accumulation in neurological conditions. Environmental factors include malnutrition, contaminated water, food, and beverages, occupational/hazardous exposures, and medical procedures. Individual metal ions have specific physiological function(s) as co-factors for many essential vitamins and proteins. High incidences of neurodegenerative diseases, such as ALS, AD, and PD, have been observed in employees in the automobile and paint industries and in other metal-utilizing factories [11,12]. Recent studies suggest that various genetic factors predispose neurons to enhanced metal toxicity. These include alterations in metal mobility or uptake across the blood-brain barrier and metal storage proteins in the brain, including lactoferrin and ferritin/transferrin. Friedreich’s ataxia, a genetic disorder of Fe metabolism, is caused by insufficient level of the Fe chaperone, frataxin, which leads to dysregulation of Fe trafficking in mitochondria and to mitochondrial genome damage by Reactive Oxygen Species (ROS) [13–16]. Similarly, abnormal Fe metabolism is responsible for the etiopathogenesis of hereditary ferritinopathy [17]. Another well-known, autosomal recessive disorder, Wilson’s disease, arises from a lethal mutation in the *ATP7B* gene, which encodes a copper (Cu) transporter, leading to supra-physiological, accumulation of free Cu in the brain and liver [18]. Aging is another contributor to chronic accumulation of brain metals [19]. Fe(III), Cu(II), and Zn(II) ions play critical roles in the gradual progression of AD and PD in an age-dependent manner by stabilizing misfolded amyloid beta sheets [20,21].

Thus, complex interactions between genetic predisposition and environmental/dietary influences appear to induce accumulation of free metal ions in the brain.

## Molecular basis of metal toxicity

**Figure 1:**
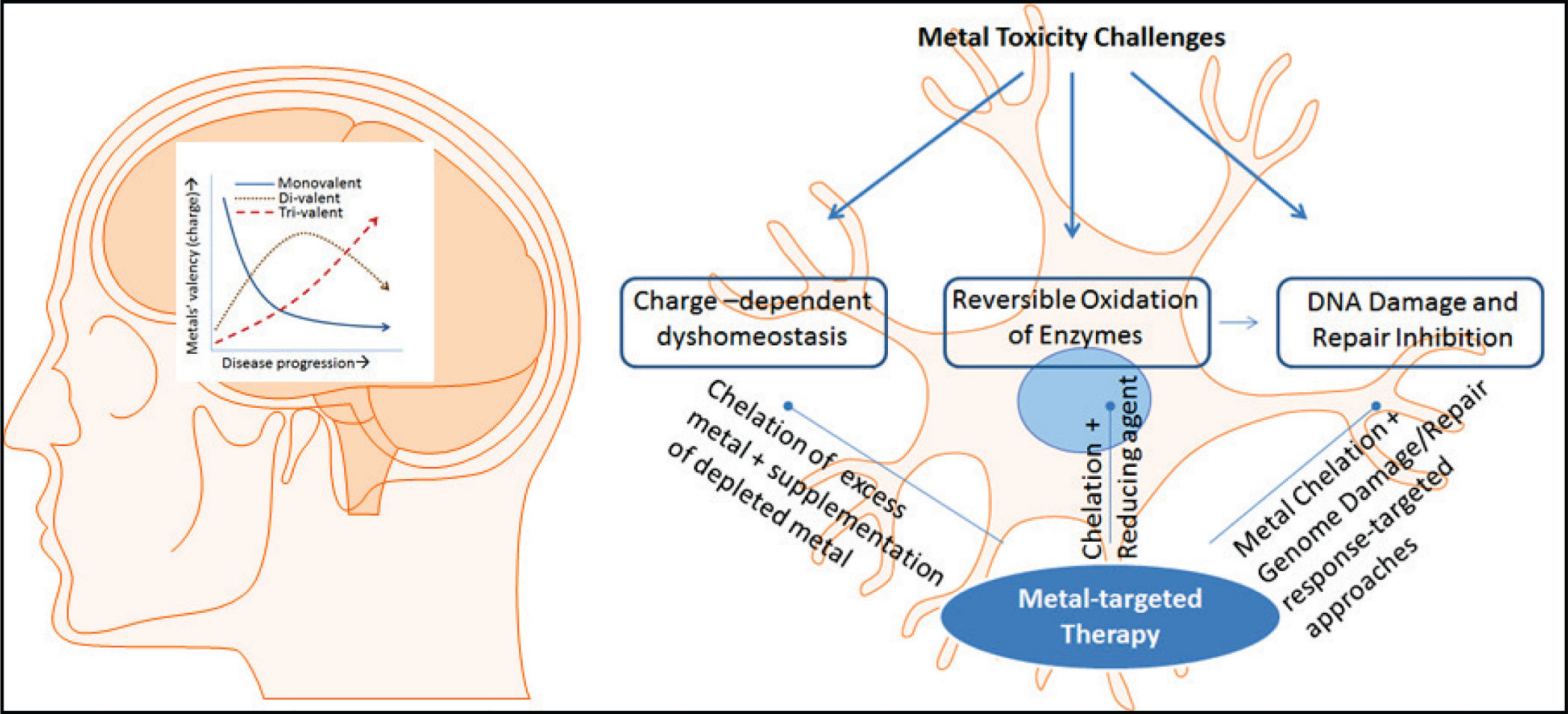
Molecular basis for metal neurotoxicity and its potential as a therapeutic target. Studies suggest a charge-based dyshomeostasis of metals in neurons affected by degenerative diseases. Typically, trivalent metals increase in late-stage AD, whereas divalent metal ions increase in early AD. The increase in metal ions could reversibly inhibit DNA repair enzymes, inducing genomic damage. Metal chelation therapy should address these challenges based on recent molecular understanding of the phenomenon.

As schematically represented in Figure 1, metal dyshomeostasis is deleterious to human cells. The intracellular and extracellular levels of metals are tightly regulated through a complex network. Excessive concentration of non-sequestered metal salts could cause cellular toxicity and pathological damage. In addition to altering the membrane potential, particularly in neurons, metal ions can bind to and affect the activity of proteins/enzymes and nucleic acid, which may cause cytotoxicity. In addition, the major cause of oxidative toxicity from transition metals is the generation of ROS, the most pervasive oxidant in cells [22–25]. In addition, many heavy metals, such as cadmium and lead, are also pro-oxidant and highly toxic. These could cause membrane depolarization by blocking calcium-ion influx and cell death [26–28].

### Individual metal accumulation versus metal homeostatic imbalance

As already stated, metal content is tightly regulated under physiological conditions in the normal brain through sequestration by storage proteins (e.g., ferritin, transferrin, and ceruloplasmin). The stored metals are released only in response to metabolic needs. Abnormal sequestration leading to metal overload is a common feature of neuronal pathologies; remarkably, studies have revealed unique charge-dependent changes in brain metal homeostasis with the progression of disease severity in AD and PD [29]. For example, the level of divalent Fe(II) or Cu(II) increases in the brain during the early phase of AD [30]. Interestingly, in AD and PD cases with no evident dietary metal exposure, the overall brain metal burden was found unaltered; instead, there is a charge-dependent redistribution of specific metals in the affected brain regions. For example, increase in Fe in PD patients, is simultaneously associated with decrease in Zn [5]. This may imply that the impact of an increase or decrease of an individual metal is not restricted to that metal alone, but causes a more dramatic overall homeostatic imbalance of metals, presumably due to loss of regulation of metals across cell membranes. This may be important for formulating metal chelation therapy, which should include supplementation of the depleted metal, in addition to chelating the increased metal ions.

It is noteworthy that in contrast to AD and PD, for other neurological disorders and stroke, there are limited data regarding trace metal homeostasis or inter-elemental relationships in the brain. A comprehensive database for the pathological dyshomeostasis of metals is critical for early diagnosis and for improving our understanding of the role of metals in neurotoxicity.

### Metals cause genomic damage directly and via generation of ROS

Oxidative genomic damage is the most common type of damage caused by pro-oxidant metals due to ROS generated via Fenton and Haber-Weiss reactions. In addition, some heavy metals including essential metals produce DNA damage via direct binding and cause strand breaks [31,32]. Both ROS and metals induce a multitude of oxidative modifications in DNA bases and sugar moieties, including DNA strand breaks. Persistent accumulation of this damage could lead to secondary double-strand breaks, which are the most toxic genomic damage [33,34].

### Inhibition of genome repair machinery by metals

While marked increase in genomic damage is observed in a majority of neurodegenerative diseases, the neurons also show decreased ability to repair the damage [35,36]. Moreover, the lack of a direct correlation between repair deficiencies and expression of repair enzymes suggests the involvement of additional mechanisms. Repair defects induced by heavy metals have been commonly attributed to their direct binding to DNA, which interferes with the repair machinery’s ability for genome damage scanning/recognition and repair [37–39]. We recently showed that physiological levels of iron and copper salts inhibited the NEIL1/2 enzymes, two key components of the oxidized DNA base repair machinery (via base excision repair or BER) [6,37,40–43]. Inhibition of these enzymes is due to metal binding to themselves rather than to DNA, which involves reversible oxidation of cysteine residues in the enzymes. Furthermore, these metal ions disrupt protein-protein interactions during BER, which is critical for coordinating the complete repair at the chromatin level. Thus our studies, in contrast to previous observations, suggest that metal-induced defects in genome repair is caused by direct binding of redox metals to specific repair proteins, which affects their redox state and structure/function. It is likely that persistent accumulation of genomic damage could elicit inflammatory responses (i.e., microglia activation), further contributing to neuronal dysfunctions [6].

## Metal-targeted therapies: Past and current challenges

Metal chelation therapy has been explored as a strategy to eliminate excess metal ions from the body. This treatment has had mixed success due to challenges intrinsically associated with chelation and the inherent complexity of metal dynamics in the body.

Some metal chelators successfully reduce metal build-up in animals and in *in vitro* models. Although chelators were shown to reduce metal accumulation in the humans in clinical trials, several challenges prevented their broad application. First, most available chelating compounds fail to cross the Blood-Brain Barrier (BBB). Desferrioxamine B (DB), an iron chelator, was one of the first metal chelators used in AD patients, where it caused a significant decline in amyloid plaque levels and decreased the cognition deterioration rate [44]. However, DB caused anemia over time [45]. Clioquinol (CQ), another metal chelator, was reported to restore metal homoestasis in several animal models of neurodegeneration and in AD patients [29,45,46]. CQ efficiently crosses the BBB, preferentially binds Cu(II) and Zn(II), and inhibits amyloid-β deposition [47]. However, a CQ derivative, 8-hydroxyquinoline (8-OHQ; also named PBT2; Prana Biotechnology), failed in a phase III trial due to non-significant clearance of β-amyloid plaques in patients with mild AD (Product News, J Gerontol. Nurs. 40, 5–6, 2004).

Magnetic resonance imaging data from phase II clinical trials indicated that deferiprone (DFP), a metal chelator used to treat thalassemia patients, significantly decreases Fe levels in the Substantia Nigra (SN) in patients with early or late PD [48]. However, after 24-month treatment, the chelation benefits of DFP disappeared, and Fe deposition reappeared in the SN. These data suggest that a readjusted treatment time should be considered for long-term benefits [48]. In contrast to other chemical chelators, DFP alleviates Fe accumulation by donating chelated Fe to unsaturated transferrin, and allows balanced retention and chemical redistribution of Fe in the body [49].

In an investigation using a mouse stroke model, the ferrous chelator 2,2'-bipyridine was shown to be effective in reducing brain injury following Intra Cerebral Hemorrhage [ICH] and ischemia [50,51]. Although this chelator appears to be promising for preventing brain injury after stroke, recent reports suggest that bipyridine does not prevent iron-induced damage in three ICH rat models [52]. Thus, its efficacy as a therapeutic agent remains unclear.

Natural metal chelators, including curcuminoids and catechins, are predominant components of the rural Asian diet and are believed to be highly beneficial for combating neurotoxicity [43]. Curcumin is the most popular curcuminoid, present in turmeric and known for its unique flavor, has a broad spectrum of pharmacological properties. In recent years, this traditional Indian spice has gained attention for its ability to bind metals and protect neurons. For example, curcumin protects hippocampal neurons against Pb- and Cd-induced lipid peroxidation [53].

Catechins comprise another class of potential metal chelators commonly found in green tea, berries, cocoa, and onions. Epigallo Catechingallate (EGCG) is a common catechin explored for its chemo-protective and neuroprotective functions. Chemically, EGCG possesses iron-chelating properties, by possibly neutralization of ferric iron and formation of redox-inactive iron in neuronal cells [1]. Although the therapeutic potential of natural compounds for acute metal toxicity needs further investigation, their inclusion in a balanced diet could provide a cost-effective strategy for reducing the oxidative burden in patients with neurodegenerative disorders or stroke.

## Challenges and future perspectives

There is compelling evidence linking metal toxicity to neuronal dysfunction. In addition, there has been an enormous increase in our understanding of the molecular basis of metal neurotoxicity. Nonetheless, current metal-targeted therapeutic approaches remain to be proven effective. Further, antioxidant therapies have not been as effective as expected. This underscores the need to explore new approaches to unraveling the bases for neuronal pathobiology. Current molecular studies have not effectively improved our ability to rationally apply metal-targeting-based therapeutic approaches. We suggest that recent basic discoveries on metal biochemistry may help develop new approaches for enhancing efficacy of metal chelation therapy. For instance, intracellular metal dyshomeostasis involving auto-depletion of specific metal ions is a common occurrence after individual metal overload; thus, metal chelation strategies should include supplementation of depleted metals. Furthermore, because of reversible oxidation of cysteine residues in various proteins, including the key genome repair enzymes NEIL1 and NEIL2 by pro-oxidant metals [37,40], metal chelation could be combined with specific reducing factors [6,29,43]. Thus, the recent advances discussed here underscore the need to revisit the role of metal toxicity in neurological diseases and stroke in order to develop new therapeutic strategy.
